# PD101 Evaluating The Budget Impact Of Introducing RefluxStop™ As A Novel Treatment Option For Gastroesophageal Reflux Disease In Italy

**DOI:** 10.1017/S0266462324003465

**Published:** 2025-01-07

**Authors:** Sam Harper, Muralikrishnan Kartha, Stuart Mealing, Maurizio Pavanello, Luigi Bonavina

## Abstract

**Introduction:**

In Italy, the first-line treatment for gastroesophageal reflux disease (GERD) is proton pump inhibitors (PPIs). Other options considered after PPI failure include Nissen fundoplication and magnetic sphincter augmentation. RefluxStop is a novel device for treating GERD. The aim of this study was to evaluate the budget impact of introducing RefluxStop into the Italian National Health Service (SSN).

**Methods:**

The analysis, which used a five-year time horizon, one-year cycle length, and Italian payer perspective, was developed in line with ISPOR recommendations. Estimates for the Italian population and growth projections, GERD prevalence and incidence rates, and rates of anti-reflux surgery were obtained from relevant literature sources. The current market share of those requiring surgery was based on market research by Implantica (Zug, Switzerland). The cost of treatment was based on an adaptation of an economic model from the UK applied to the Italian setting. Unit costs were derived from available literature and diagnosis-related groups averaged across five Italian regions. Extensive sensitivity analyses involving key model inputs were also conducted.

**Results:**

The overall one-year, three-year, and five-year financial impacts of introducing RefluxStop were EUR 242,641, EUR 710,651, and EUR 1,004,946 per year, respectively, corresponding to 0.063 percent, 0.197 percent, and 0.316 percent annual increases in overall Italian SSN expenditure for GERD. Use of more optimistic and pessimistic market uptake and surgery rates (i.e., doubled and halved) for RefluxStop led to one-year increases in Italian SSN expenditure of 0.126 percent and 0.032 percent and five-year increases of 0.636 percent and 0.155 percent, respectively. In our study, introducing RefluxStop avoided 95 surgical failures, 11 reoperations, and 64 endoscopic esophageal dilatations over five years.

**Conclusions:**

The introduction of RefluxStop is likely to provide substantial benefits for patients with GERD at the cost of a marginal budget impact on the Italian healthcare system. Though Italian health care is organized at a regional level, this study is an example of a health technology assessment using budget impact analysis at a national level.

